# A retrospective observational study of changes in uterine fibroid blood flow and fibroid diameter after administration of gonadotropin‐releasing hormone agonists and antagonists using superb microvascular imaging

**DOI:** 10.1111/jog.16361

**Published:** 2025-07-03

**Authors:** Yudai Tanaka, Hiroaki Fujita, Reiko Kitayama, Minako Katano, Eri Nakano, Naoki Yoshikawa, Aya Shiraiwa, Kana Hirayama, Akane Takaya, Reiko Numazaki, Yuki Furusawa, Yoshihiro Nishijima, Eri Obiya

**Affiliations:** ^1^ Medical Park Shonan Fujisawa City Kanagawa Japan

**Keywords:** fibroid blood flow, leuprorelin (leuprolide), relugolix, superb microvascular imaging, uterine fibroids

## Abstract

**Aim:**

The relationship between the changes in fibroid blood flow and fibroid diameter during the administration of GnRH agonists and antagonists was examined using superb microvascular imaging (SMI).

**Methods:**

Changes in the maximum fibroid blood flow and diameter at weeks 0, 2, 4, and 8 were compared between the relugolix, a GnRH antagonist, and leuprolide, a GnRH agonist, groups.

**Results:**

Data were collected for 16 fibroids from 12 patients in the relugolix group and 12 fibroids from 9 patients in the leuprorelin group. A significant decrease was observed in the fibroid diameter at 8 weeks in the relugolix group, but no significant change was observed at any time point in the leuprorelin group. In the relugolix group, a significant correlation were observed between changes in blood flow at 2 weeks and fibroid diameter changes at 2 and 4 weeks, and between changes in blood flow at 4 weeks and fibroid diameter changes at 4 weeks. No significant correlation was observed in the leuprorelin group.

**Conclusion:**

In the treatment of uterine fibroids with relugolix, a reduction in fibroid blood flow assessed by SMI may suggest a subsequent reduction in fibroid size. These results provide useful insights when making the decision whether or not to continue relugolix administration.

## INTRODUCTION

Uterine fibroids are common benign tumors found in women of reproductive age,^1^ and their prevalence varies by country and race, ranging from 4.5% to 68.6%.^2^ Recent ultrasound studies have shown that more than 80% of African‐American women and nearly 70% of Caucasian women have uterine fibroids.^3^ Uterine fibroids affect patients' quality of life, with symptoms including pelvic and abdominal pain, irregular bleeding, dysmenorrhea, and infertility.^1^, ^4^

Treatment for uterine fibroids mainly consists of pharmacotherapy, uterine artery embolization (UAE), and surgery; treatment should be individualized based on the size and number of tumors, severity of symptoms, and need to preserve the uterus.^1^ GnRH agonists (GnRH‐ago) and antagonists (GnRH‐ant) are the most commonly used agents in pharmacotherapy.^1^ Both agents potentially inhibit gonadotropin secretion from the pituitary gland and suppress estrogen and progesterone at the level of menopause, subsequently improving the symptoms and reducing the size of uterine fibroids.^4^ GnRH‐ago causes a flare‐up in which the secretion of sex hormones increases in the early stages of administration, while GnRH‐ant does not cause a flare‐up because it directly suppresses gonadotropins.^5^

One of the pathological characteristics of uterine fibroids is the presence of a capsule rich in blood vessels surrounding the fibroids, called a vascular capsule or pseudocapsule, which is thought to be involved in abnormal bleeding in uterine fibroids.^6^, ^7^, ^8^, ^9^ Although blood vessels from the vascular capsule supply blood to fibroids,^6^ there are few large blood vessels inside the fibroid. Moreover, the diameter of vessels inside fibroids is reported to be smaller than the capillaries of the normal endometrium.^10^, ^11^ Thus, uterine fibroids have the structural characteristic of being surrounded by a blood‐rich vascular capsule, yet with low blood flow inside.^6^ It has been reported that there is a correlation between uterine blood flow and its size,^9^, ^12^, ^13^ and the fibroid blood flow may be an indicator of treatment efficacy. However, as mentioned above, blood flow in the fibroid tissue itself is too low to detect by conventional color Doppler ultrasound. Thus, a more sensitive technique has been required to evaluate blood flow inside the uterine fibroid.

Superb microvascular imaging (SMI) is a new Doppler ultrasound measurement technique that can detect low‐velocity blood flow in small blood vessels without the use of contrast agents.^14^ Samanci et al. reported that the treatment effect of UAE on uterine fibroids is higher in fibroids with hypervascularity than in those with hypovascularity and that the treatment success rate can be predicted based on vascularity.^15^ The sensitivity, specificity, positive predictive value, negative predictive value, and accuracy of treatment success for hypervascular fibroids were 36.8%, 100.0%, 100.0%, 42.0%, and 57.1%, respectively, when vascular status was evaluated by Power Doppler ultrasonography, whereas these values increased to 73.6%, 100.0%, 100.0%, 64.0%, and 82.1%, respectively, when evaluated by SMI.^15^ In general, Color Doppler flow imaging is difficult to describe blood flow when the vessel diameter is small (<1 mm) and the blood flow velocity is slow (3–5 cm/s).^14^ This is due to the difficulty in distinguishing between low‐velocity blood flow and artifacts caused by tissue movements such as breathing.^14^ On the other hand, SMI can depict slow blood flow in even small vessels (<1 mm) by analyzing noise and discriminating between tissue motion artifacts and actual blood flow.^14^ Although blood flow inside uterine fibroids is rather sparse, SMI can clearly detect it and might make it possible to predict the effectiveness of pharmacotherapy. In this study, we used SMI to observe blood flow in uterine fibroids treated with GnRH‐ago or GnRH‐ant, the standard therapeutic agents for uterine fibroids, and evaluated the relationship between changes in blood flow and their therapeutic effects.

## MATERIALS AND METHODS

### Purpose

The objectives of this study were (1) to compare the depiction of uterine fibroid blood flow using color Doppler and SMI, (2) to evaluate the effects of GnRH‐ago and GnRH‐ant on fibroid blood flow and fibroid diameter, (3) to compare the improvement of uterine fibroids (cumulative number of times in which fibroid blood flow and fibroid diameter decreased) between the GnRH‐ago and GnRH‐ant groups, and (4) to examine the possibility of predicting treatment outcomes based on changes in fibroid blood flow undergoing GnRH‐ago or GnRH‐ant treatment.

### Patients

This retrospective study included patients aged 20 years or older who received treatment with relugolix (40 mg orally administered once a day), a GnRH‐ant, or leuprorelin (subcutaneous injection of 1.88 mg every 4 weeks), a GnRH‐ago, for uterine fibroids for 8 weeks or more at Medical Park Shonan between April 2023 and October 2023.

### Methods

Patients' characteristics including age, number of gravidity and parity, number of fibroids, fibroid diameter, fibroid type, fibroid blood flow, and symptoms were collected. To compare the depiction capabilities of color Doppler and SMI, we compared the blood flow in and around the fibroids in the same cases. To evaluate the effects of GnRH‐ago and GnRH‐ant on fibroid blood flow and fibroid diameter, the changes in fibroid blood flow after administration of relugolix and leuprorelin were observed in one case each, and the changes in fibroid blood flow and diameter in both groups were compared using spaghetti plots for each case, as well as the mean and median values for all cases. Improvement was defined as both a reduction in the maximum diameter of the fibroid and a decrease in blood flow detected by SMI. To compare the cumulative mean number of improvements between the GnRH‐ago and GnRH‐ant groups, the cumulative mean number of improvements was evaluated in the relugolix and leuprorelin groups. To examine the possibility of predicting treatment outcomes based on changes in fibroid blood flow undergoing GnRH‐ago or GnRH‐ant treatment, the correlation between changes in fibroid blood flow and changes in maximum fibroid diameter at 2, 4, and 8 weeks after administration was evaluated in the relugolix and leuprorelin groups, respectively. In this study, a transvaginal probe was primarily used, and a transabdominal probe was used when a transvaginal approach was difficult (e.g., giant fibroids, etc.).

### Superb microvascular imaging

An Aplio i800 (Canon Medical Systems Corporation) transvaginal or transabdominal probe was used to image the fibroids. In patients with multiple fibroids, up to three fibroids were imaged and analyzed. The maximum diameter of the uterine fibroid was measured in the 2D B‐mode, and the region of interest in the SMI mode was set to include the entire fibroid. A probe scan was performed, and a video was saved. The maximum fibroid diameter was measured by selecting the frame with the largest diameter from the saved B‐mode videos. For blood flow analysis, the frame with the greatest blood flow was visually selected from the saved SMI mode video, the boundary of the uterine fibroid was traced using the vascular index function, and the area of the blood flow signal was calculated. The SMI frequency, gain, flow velocity, and various filters were all standardized for all cases. For cases with a lot of noise, the gain was adjusted in B‐mode to reduce noise and evaluate blood flow. If motion artifacts that couldnot be reduced were observed, that area was excluded by the vascular index function of SMI.

### Statistical analysis

Student's *t*‐test was used to compare continuous variables, and the Chi‐square test was used to compare categorical variables. The 95% confidence interval (CI) was calculated to determine differences in the rate of change of the fibroid blood flow and maximum fibroid diameter between the groups. The significance of changes in fibroid blood flow and fibroid diameter was determined based on whether the 95% CI crossed zero.^16^ The correlation between the change in fibroid blood flow and the change in fibroid diameter was evaluated using Pearson's correlation coefficient. Regarding the efficacy of the relugolix and leuprorelin groups, the cumulative mean number of improvements was calculated. The mean number of improvements is the number of improvement events (reduction in fibroid blood flow and fibroid diameter) that occurred in each group every 2 weeks divided by the number of cases in each group (it means mean number of improvement events per case). The cumulative mean number of improvements is calculated by accumulating the mean number of improvement events. The cumulative mean occurrence of responses every 2 weeks from week 0 to week 8 was calculated according to the method of Lawless et al.,^17^ and a two‐sided test based on the normal approximation was performed under the null hypothesis that the cumulative mean occurrences in the two groups were equal. 95%CI and the cumulative mean number of improvements were calculated using R version 4.4.1 (RStudio, PBC, Boston, MA, USA). Student's t‐test, Chi‐square test, and Pearson's correlation coefficient were calculated using Microsoft Excel for Microsoft 365 (Microsoft Corp., WA, USA).

## RESULTS

### Patient characteristics

Data were collected for 16 fibroids from 12 patients in the relugolix group and 12 fibroids from nine patients in the leuprorelin group. All patients had some symptoms due to uterine fibroids. There were no significant differences between the two groups in terms of age, number of gravidity and parity, number of fibroids, fibroid diameter, fibroid blood flow, fibroid type, or symptoms (Table 1). A transabdominal probe was used in two patients in the relugolix group, and a transvaginal probe was used in all other patients.

**TABLE 1:** Demographic and baseline characteristics.

	Relugolix (*n* = 12)	Leuprorelin (*n* = 9)	*p*
Age, mean ± SD	44.2 ± 5.37	45.0 ± 6.42	0.763
Gravidity, mean ± SD	1.0 ± 1.73	1.2 ± 1.39	0.753
Parity, mean ± SD	0.5 ± 0.88	1.2 ± 1.39	0.131
Total number of fibroids in each group, *n*	16	12	
Number of fibroids, *n* (%)			
1	8	6	0.676
2	4	3	
Size of fibroids (mm)			
Mean ± SD	64.8 ± 34.61	58.7 ± 25.25	0.613
Median (min, max)	53.5 (21.1, 120.0)	53.7 (28.3, 107.0)	
Area of blood flow (cm^2^)			
Mean ± SD	2.8 ± 3.6	1.8 ± 0.99	0.337
Median (min, max)	1.3 (0.1, 13.1)	1.7 (0.4, 4.1)	
Type of fibroids, *n*			
Intramural myoma	10	8	0.820
Submucous myoma	2	3	0.393
Subserous myoma	4	1	0.254
Symptoms, *n* (%)			
Heavy menstrual bleeding or anemia	5 (41.7%)	5 (55.6%)	0.528
Pain	4 (33.3%)	2 (22.2%)	0.577
Irregular menstrual bleeding	3 (25.0%)	1 (11.1%)	0.422
Bloating	3 (25.0%)	4 (44.4%)	0.350
Urinary frequency/feeling of residual urine	1 (8.3%)	3 (33.3%)	0.149
Infertility	1 (8.3%)	1 (11.1%)	0.830
Probe, *n*			
Transvaginal probe	10	9	0.198
Transabdominal probe	2	0	

### Imaging of blood flow around fibroids using SMI and color Doppler

**FIGURE 1:**
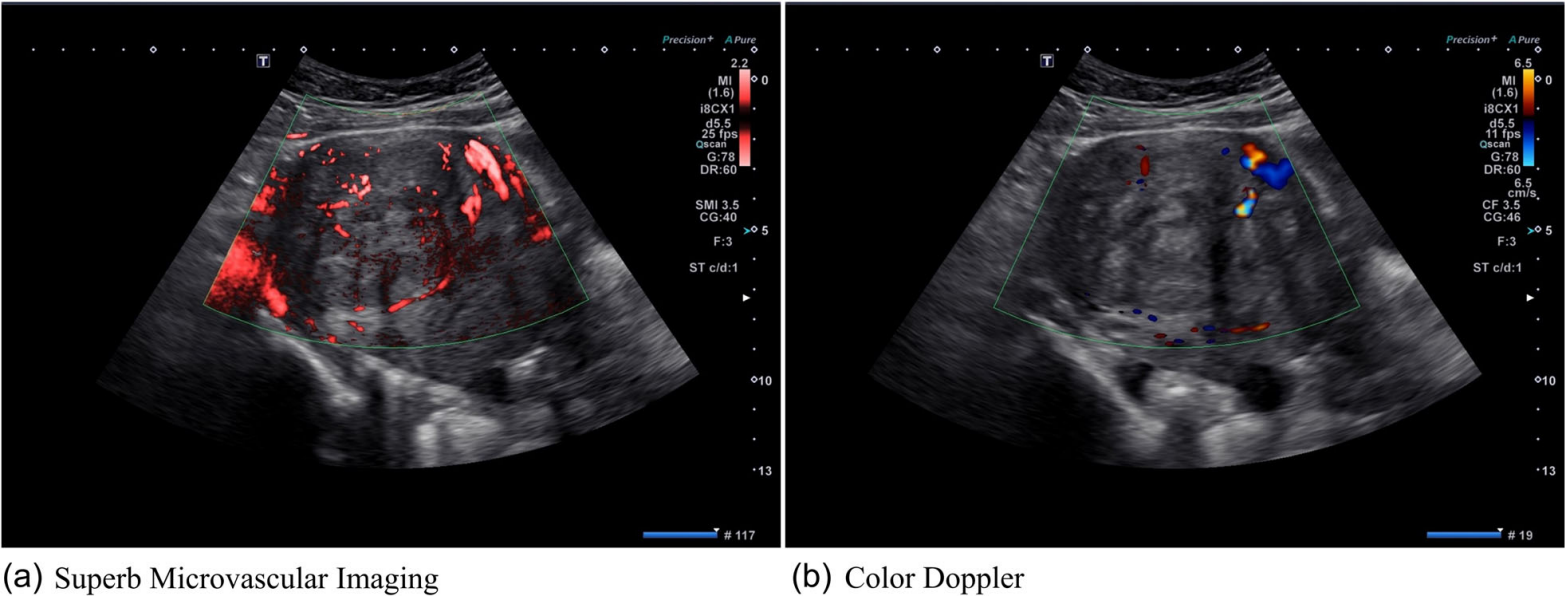
Superb microvascular imaging and color Doppler imaging. (a) Superb microvascular imaging demonstrated strong blood flow in the presumed vascular capsule surrounding the fibroid, as well as weak blood flow within the fibroid. (b) Color Doppler revealed a large blood vessel in the upper‐right corner of the fibroid; however, other small blood vessels were barely visible.

Figure 1 shows the SMI and color Doppler images obtained via transabdominal probe at the same time point in the same patient. SMI demonstrated weak blood flow within the fibroid in addition to strong blood flow in the presumed vascular capsule surrounding the fibroid (Figure 1a). Color Doppler revealed a large blood vessel in the upper‐right corner of the fibroid; however, other small blood vessels were barely visible (Figure 1b).

### Changes in blood flow in patients receiving relugolix and leuprorelin

**FIGURE 2:**
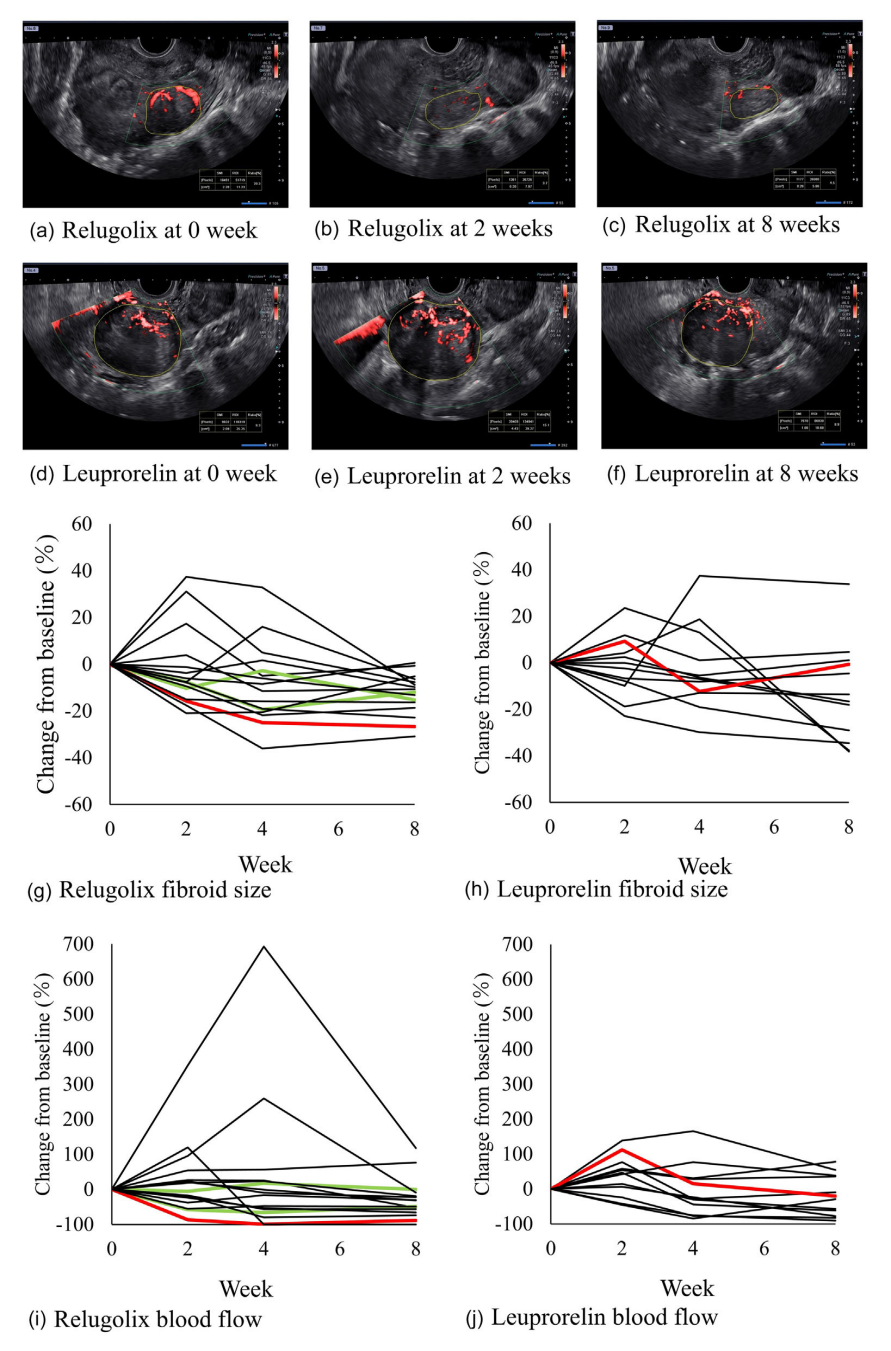
SMI images and changes in fibroid blood flow and fibroid diameter. (a)–(c) In patients receiving relugolix, relatively large blood vessels were observed within the fibroids at 0 week, but no large blood vessels were observed at 2 weeks. At 8 weeks, blood vessels flowing into and out of the fibroids were observed; however, almost no blood vessels were observed within the fibroids. (d)–(f): In patients receiving leuprorelin, compared with 0 week, there was a relative increase in blood flow at 2 weeks and a decrease in blood flow at 8 weeks. The changes in fibroid blood flow and fibroid diameter in cases where relugolix and leuprorelin were administered in (a)–(f) are shown by red lines in (g)–(j). (g) and (h): Compared with the leuprorelin group, there were relatively more cases in the relugolix group in which fibroid diameter decreased from 2 weeks after administration. A transabdominal probe was used in two patients in the relugolix group, and a transvaginal probe was used in all other patients. The green lines show the changes in fibroid diameter and fibroid blood flow in two patients in the relugolix group who received a transabdominal probe (g) and (i). The changes in blood flow and fibroid diameter in cases using a transabdominal probe were similar to those in cases using a transvaginal probe. (i), (j): The leuprorelin group showed relatively greater variability in changes in fibroid size and fibroid blood flow than the relugolix group.

Figure 2a–f shows the SMI images at weeks 0, 2, and 8 for one patient each administered relugolix and leuprorelin. In the patient receiving relugolix, relatively large blood vessels were observed within the fibroid at week 0 (Figure 2a), but no large blood vessels were observed at week 2 (Figure 2b). At week 8, blood vessels flowing into and out of the fibroids were observed; however, almost no blood vessels were observed within the fibroids (Figure 2c). In the patient receiving leuprorelin, compared with week 0 (Figure 2d), there was a relative increase in blood flow at week 2 (Figure 2e) and a decrease at week 8 (Figure 2f). The changes in fibroid blood flow and fibroid diameter in cases where relugolix and leuprorelin were administered in Figure 2a–f are shown by red lines in Figure 2g–j. In patients receiving relugolix, a decrease in blood flow within the fibroids was observed 2 and 8 weeks after administration (Figure 2i), but no clear decrease in blood flow was observed in the leuprorelin group (Figure 2j).

### Changes in maximum fibroid diameter and fibroid blood flow in the relugolix and the leuprorelin groups

The rates of change in the maximum fibroid diameter and fibroid blood flow from weeks 0 to 8 in the relugolix and leuprorelin groups are shown in Table 2. The maximum diameter of the uterine fibroids relatively decreased after 2 weeks in both groups. The rate of change in the maximum uterine diameter significantly decreased at 8 weeks in the relugolix group (mean rate of change [95%CI]: −12.8% [−17.4, −8.1]), while no significant changes were observed in the leuprorelin group. There was no clear difference in the rate of change in blood flow between the two groups. Spaghetti plots of percent change in fibroid diameter and blood flow are shown in Figure 2g–j. The green lines showed the changes in fibroid diameter and fibroid blood flow in two patients in the relugolix group who received a transabdominal probe (Figure 2g–i). The changes in blood flow and fibroid diameter in cases using a transabdominal probe were similar to those in cases using a transvaginal probe. Compared with the leuprorelin group, there were relatively more cases in the relugolix group in which fibroid diameter decreased from 2 weeks after administration (Figure 2g, h). In addition, the leuprorelin group showed relatively greater variability in changes in fibroid blood flow and fibroid size than the relugolix group (Figure 2g–j).

**TABLE 2:** Rate of changes in maximum fibroid diameter and fibroid blood flow.

	Relugolix (*n* = 12)	Leuprorelin (*n* = 9)	Inter group difference
Total number of fibroids, *n*	16	12	
Rate of change in maximum diameter			
Week 2			
Mean [95%CI]	−2.1% [−11.0, 6.9]	−1.4% [−9.7, 6.8]	−0.7% [−13.0, 11.6]
Median (min, max)	−6.6% (−20.9, 37.4)	−1.2% (−22.9, 23.6)	
Week 4			
Mean [95%CI]	−8.5% [−17.5, 0.5]	−2.6% [−14.1, 8.9]	−6.0% [−19.8, 7.9]
Median (min–max)	−10.0% (−36.0, 32.9)	−6.7% (−29.8, 37.4)	
Week 8			
Mean [95%CI]	−12.8% [−17.4, −8.1]	−12.8% [−26.2, 0.7]	−0.05% [−12.2, 12.1]
Median (min–max)	−11.3% (−30.8, 0.6)	−15.1% (−38.2, 33.8)	
Rate of change in blood flow			
Week 2			
Mean [95%CI]	25.5% [−29.5, 80.5]	36.5% [−0.2, 73.1]	−8.2% [−75.2, 59.0]
Median (min–max)	6.7% (−86.8, 353.6)	44.4% (−84.4, 165.8)	
Week 4			
Mean [95%CI]	34.0% [−70.1, 138.2]	−3.9% [−50.0, 42.3]	40.7% [−81.0, 162.4]
Median (min–max)	−13.0% (−100.0, 692.9)	−25.9% (−84.4, 165.8)	
Week 8			
Mean [95%CI]	−28.4% [−58.6, 1.8]	−18.5% [−55.3, 18.4]	−7.2% [−52.9, 38.5]
Median (min–max)	−40.3% (−100.0, 117.9)	−24.7% (−90.6, 77.8)	

### Cumulative mean number of improvements in the relugolix and the leuprorelin groups

**FIGURE 3:**
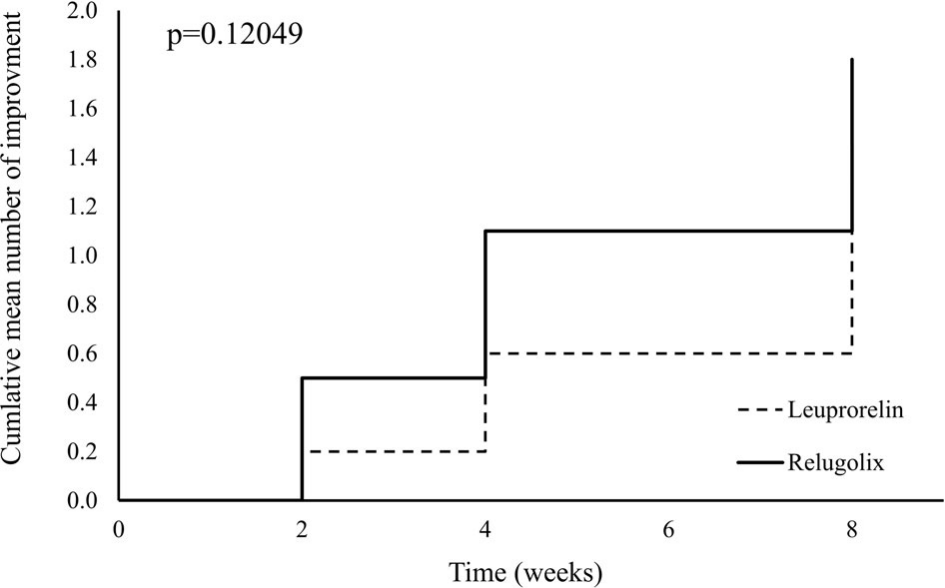
Cumulative mean number of improvements in the Relugolix and Leuprorelin groups. The mean number of improvements is the number of improvement events (reduction in fibroid blood flow and fibroid diameter) that occurred in each group every 2 weeks divided by the number of cases in each group (it means mean number of improvement events per case). The cumulative mean number of improvements is calculated by accumulating the mean number of improvements. Although there was no significant difference between the two groups in the cumulative mean number of improvements, the relugolix group tended to show earlier improvement.

Although there was no significant difference between the two groups in the cumulative mean number of improvements, the relugolix group tended to show more rapid improvement than the leuprorelin group (Figure 3).

### Correlation between changes in fibroid blood flow and changes in fibroid diameter in the relugolix and the leuprorelin groups

**FIGURE 4:**
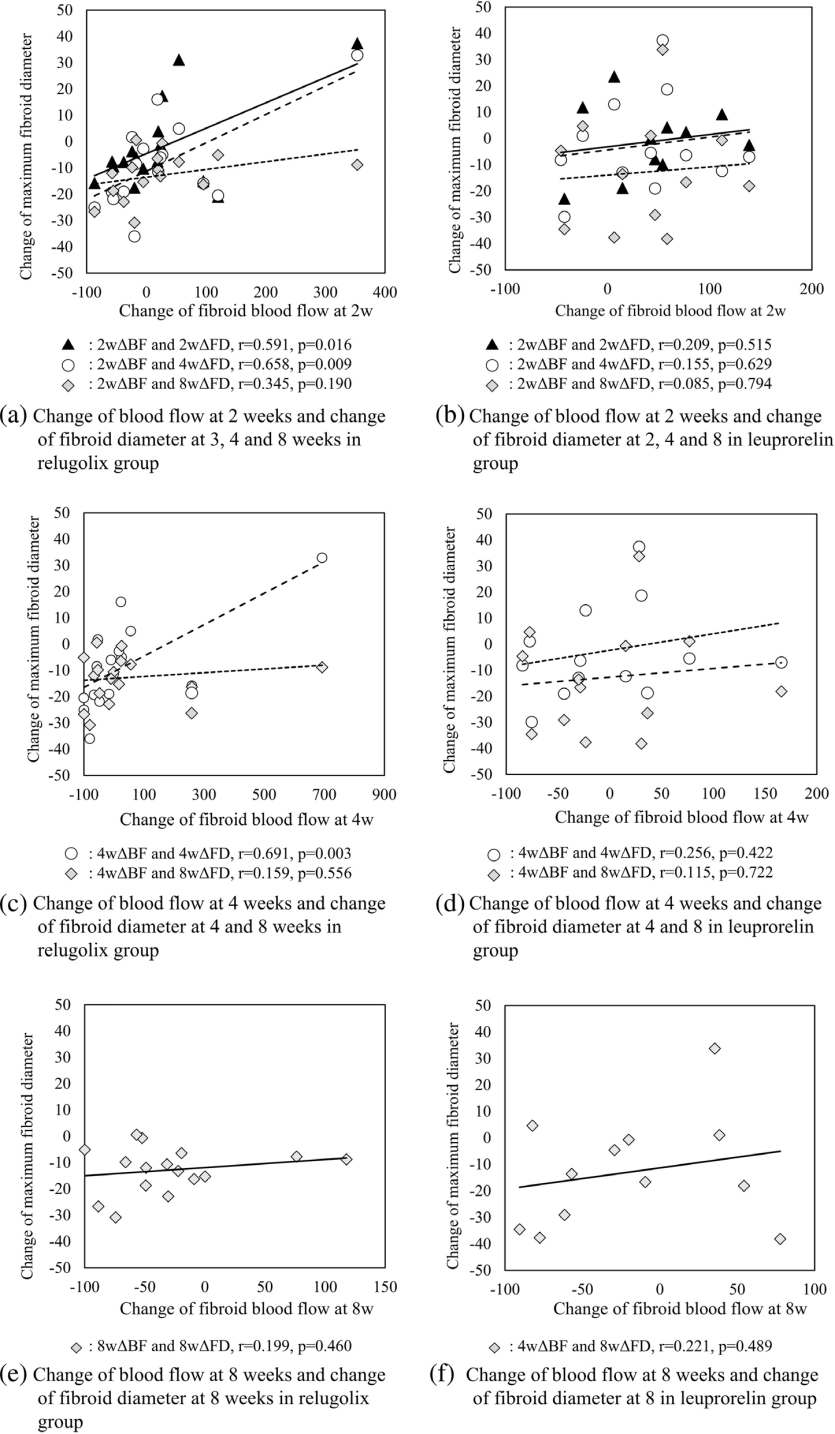
Correlation between changes in blood flow and changes in maximum diameter of fibroids. 2wΔBF: Changes in fibroid blood flow from 0 to 2 weeks, 4wΔBF: Changes in fibroid blood flow from 0 to 4 weeks, 8wΔBF: Changes in fibroid blood flow from 0 to 8 weeks, 2wΔFD: Changes in fibroid diameter from 0 to 2 weeks, 4wΔFD: Changes in fibroid diameter from 0 to 4 weeks, 8wΔFD: Changes in fibroid diameter from 0 to 8 weeks. (a) and (b): Correlation between change in fibroid blood flow from 0 to 2 weeks and change in fibroid diameter from 0 to 2, 4, and 8 weeks in both groups. In the relugolix group, a significant correlation was observed between the change in blood flow at 2 weeks and the change in fibroid diameter at two and 4 weeks, but no significant correlation was observed at 8 weeks. In the leuprorelin group, no significant correlation was observed at any time point. (c) and (d): Correlation between change in fibroid blood flow from 0 to 4 weeks and change in fibroid diameter from 0 to 4, and 8 weeks in both groups. In the relugolix group, a significant correlation was observed between the change in blood flow at 4 weeks and the change in fibroid diameter at 2 and 4 weeks, but no significant correlation was observed at 8 weeks. In the leuprorelin group, no significant correlation was observed at any time point. (e) and (f): Correlation between change in fibroid blood flow from 0 to 8 weeks and change in fibroid diameter from 0 to 8 weeks in both groups. No significant correlation was observed in both groups.

Figure 4a–f shows the correlation between the change in fibroid blood flow and the changes in maximum fibroid diameter. In the relugolix group, a significant correlation was observed between the change in blood flow at week 2 and the changes in fibroid diameter at week 2 (*r* = 0.591, *p* = 0.016) and 4 (*r* = 0.658, *p* = 0.009), and the change in blood flow at week 4 and the change in fibroid diameter at week 4 (*r* = 0.691, *p* = 0.003) (Figure 4a and c). No significant correlation was observed between the two at other time points in the relugolix group (Figure 4c and e). In the leuprorelin group, no significant correlation was observed between changes in blood flow and fibroid diameter at any time point (Figure 4b, d, f).

## DISCUSSION

Changes in uterine fibroid blood flow and fibroid diameter after the administration of GnRH‐ago and GnRH‐ant were evaluated using SMI. Consistent with previous findings,^6^, ^10^, ^11^ blood flow was found to be abundant in the vascular capsule surrounding the fibroids, whereas blood flow within the fibroids was low. Compared with color Doppler imaging, SMI was able to depict areas of low blood flow within fibroids. Previous studies have shown that SMI is useful for depicting areas with low blood flow, such as the breasts, pediatric testes, neonatal brain parenchyma, and low‐grade inflammation in arthritis.^14^, ^18^, ^19^, ^20^, ^21^ This study demonstrates that SMI can depict the inside of fibroids, which have low blood flow.

During the 8‐week observation period in this study, a significant decrease in the maximum diameter of the fibroids was observed at week 8 in the relugolix group, whereas no significant decrease was observed in the leuprorelin group. We speculate that the difference comes from whether each agent has flare‐up or not; GnRH‐ago does, while GnRH‐ant does not. However, as there was no significant difference in the change in the maximum diameter of the fibroids between the two groups, the reason for the lack of significant change in the leuprorelin group may be due to the large variation between cases and the small sample size of 9 cases. Although there was no significant difference in the cumulative mean number of improvements, the relugolix group showed a relatively faster improvement than the leuprorelin group. These background factors are also speculated to be due to the property of relugolix, which directly suppresses gonadotropins and does not cause flare‐ups.

Frijlingh et al. reported a positive correlation between the number of blood vessels present in fibroids before treatment and the rate of change in the fibroid diameter after treatment.^9^ In addition, Aleem et al. reported that in the treatment of uterine fibroids, a reduction in fibroid volume was observed after a reduction in uterine blood flow.^22^ Based on these findings, we investigated whether treatment outcomes could be predicted by changes in fibroid blood flow. No correlation was observed between changes in fibroid blood flow and fibroid diameter in the leuprorelin group; however, a significant correlation was observed between changes in fibroid blood flow at 2 weeks and changes in fibroid diameter at 2 and 4 weeks and changes in blood flow at 4 weeks and changes in fibroid diameter at 4 weeks in the relugolix group. The significant correlation observed in the relugolix group is consistent with findings reported by Frijlingh and Aleem et al.^9^, ^22^ On the other hand, there was no significant correlation between the change in blood flow at 2 weeks and the fibroid diameter at 8 weeks, or between the change in blood flow at 4 weeks and the fibroid diameter at 8 weeks. This may be because the change in fibroid diameter occurs within 2 weeks after the decrease in blood flow. The reason why no correlation was observed at any time point in the leuprorelin group may have been the effects of flare‐ups and the large inter‐case variability in both changes in fibroid diameter and blood flow. Therefore, it seemed difficult to predict subsequent changes in fibroid diameter from changes in fibroid blood flow in the leuprorelin group. Our results suggest that if a decrease in fibroid blood flow is observed, further reduction in fibroid diameter may be expected and is likely to be useful information in determining whether or not to continue administering relugolix. Adverse events associated with GnRH‐ago and GnRH‐ant include hot flashes, irregular bleeding, and bone loss.^5^, ^23^ Providing information on whether to continue relugolix administration for the treatment of uterine fibroids is thought to be clinically significant in terms of avoiding these adverse events. This finding was made possible by using relugolix, a GnRH‐ant that does not cause flare‐ups, and SMI, which allows the visualization of low blood flow within fibroids. Relugolix treatment and the evaluation of fibroids using SMI are likely to contribute to advances in the treatment of uterine fibroids.

One of the strengths of this study is the rarity of evaluating SMI using GnRH‐ant in the treatment of uterine fibroids. To our knowledge, this is the first study to evaluate the relationship between fibroid blood flow and fibroid diameter during treatment with GnRH‐ant.

This study has several limitations. The number of cases in each group was 12 and 9, respectively, and there were limitations owing to the sample size. This was a retrospective observational study, and selection and confounding biases could not be excluded. The observation period of this study was 8 weeks, and longer‐term evaluation is a future task. SMI was performed by a single expert and not by multiple experts. The probes used were not consistent; 19 of the 21 patients were transvaginal and 2 were transabdominal. Although SMI can minimize artifacts, it is not perfect.^24^ Because a histological evaluation was not performed, the factors related to variations in blood flow are unknown.

In patients with uterine fibroids treated with relugolix, significant correlations were observed between changes in fibroid blood flow at 2 weeks and fibroid size at 2 and 4 weeks, as well as between both at 4 weeks evaluated by SMI. A reduction in fibroid blood flow with relugolix administration may lead to a subsequent reduction in fibroid size within 2 weeks. Evaluating changes in blood flow in fibroids using SMI may provide useful information for deciding whether to continue administering GnRH ant. On the other hand, since no correlation was observed between fibroid blood flow and changes in fibroid size after 8 weeks of administration, further study is needed to predict fibroid shrinkage with long‐term administration.

## AUTHOR CONTRIBUTIONS

**Yudai Tanaka:** Conceptualization; data curation; formal analysis; investigation; methodology; supervision; validation; writing – original draft; writing – review and editing. **Hiroaki Fujita:** Conceptualization; investigation; methodology; writing – review and editing. **Reiko Kitayama:** Investigation; resources; writing – review and editing. **Minako Katano:** Data curation; investigation; resources; writing – review and editing. **Eri Nakano:** Data curation; investigation; resources; writing – review and editing. **Naoki Yoshikawa:** Data curation; investigation; resources; writing – review and editing. **Aya Shiraiwa:** Investigation; methodology; resources; writing – review and editing. **Kana Hirayama:** Investigation; resources; writing – review and editing. **Akane Takaya:** Investigation; resources; writing – review and editing. **Reiko Numazaki:** Investigation; resources; writing – review and editing. **Yuki Furusawa:** Investigation; resources; writing – review and editing. **Yoshihiro Nishijima:** Investigation; resources; writing – review and editing. **Eri Obiya:** Investigation; resources; writing – review and editing.

## CONFLICT OF INTEREST STATEMENT

YT received speaker fee from ASKA Pharmaceutical Co. Ltd. Other authors have no conflict of interest.

## ETHICS STATEMENT

This study was conducted in accordance with the ethical principles of the Declaration of Helsinki and in compliance with the ethical guidelines for medical research involving human subjects. In this study, subjects were given the opportunity to refuse to participate in the study by opting out. This study protocol was approved by the Ethics Committee of Medical Park Shonan.

## Data Availability

This study was conducted in compliance with the Declaration of Helsinki and compliance with the ethical guidelines for medical research involving human subjects. This research protocol did not include sharing patients' data in a public repository. Hence, the data are not publicly available due to ethical restrictions. The data that support the findings of this study could be available from the corresponding author upon reasonable request.

## References

[1] Giuliani E , As‐Sanie S , Marsh EE . Epidemiology and management of uterine fibroids. Int J Gynaecol Obstet. 2020;149:3–9. 31960950 10.1002/ijgo.13102

[2] Stewart EA , Cookson CL , Gandolfo RA , Schulze‐Rath R . Epidemiology of uterine fibroids: a systematic review. BJOG. 2017;124:1501–1512. 28296146 10.1111/1471-0528.14640

[3] Baird DD , Dunson DB , Hill MC , Cousins D , Schectman JM . High cumulative incidence of uterine leiomyoma in black and white women: ultrasound evidence. Am J Obstet Gynecol. 2003;188:100–107. 12548202 10.1067/mob.2003.99

[4] Ghant MS , Sengoba KS , Recht H , Cameron KA , Lawson AK , Marsh EE . Beyond the physical: a qualitative assessment of the burden of symptomatic uterine fibroids on women's emotional and psychosocial health. J Psychosom Res. 2015;78:499–503. 25725565 10.1016/j.jpsychores.2014.12.016

[5] Osuga Y , Enya K , Kudou K , Tanimoto M , Hoshiai H . Oral gonadotropin‐releasing hormone antagonist Relugolix compared with Leuprorelin injections for uterine Leiomyomas: a randomized controlled trial. Obstet Gynecol. 2019;133:423–433. 30741797 10.1097/AOG.0000000000003141

[6] Ciarmela P , Delli Carpini G , Greco S , Zannotti A , Montik N , Giannella L , et al. Uterine fibroid vascularization: from morphological evidence to clinical implications. Reprod Biomed Online. 2022;44:281–294. 34848152 10.1016/j.rbmo.2021.09.005

[7] Frijlingh M , Juffermans L , de Leeuw R , de Bruyn C , Timmerman D , van den Bosch T , et al. How to use power Doppler ultrasound in transvaginal assessment of uterine fibroids. Ultrasound Obstet Gynecol. 2022;60:277–283. 35195311 10.1002/uog.24879 PMC9543636

[8] Walocha JA , Litwin JA , Miodoński AJ . Vascular system of intramural leiomyomata revealed by corrosion casting and scanning electron microscopy. Hum Reprod. 2003;18:1088–1093. 12721189 10.1093/humrep/deg213

[9] Frijlingh M , De Milliano I , Hehenkamp WJK , Huirne JAF . Differences in fibroid vascularity after three months of pre‐treatment with leuprolide acetate or ulipristal acetate: a pilot study. Eur J Obstet Gynecol Reprod Biol. 2020;245:186–192. 31679806 10.1016/j.ejogrb.2019.08.002

[10] Aitken E , Khaund A , Hamid SA , Millan D , Campbell S . The normal human myometrium has a vascular spatial gradient absent in small fibroids. Hum Reprod. 2006;21:2669–2678. 16807279 10.1093/humrep/del220

[11] Stirland DL , Nichols JW , Jarboe E , Adelman M , Dassel M , Janát‐Amsbury MM , et al. Uterine perfusion model for analyzing barriers to transport in fibroids. J Control Release. 2015;214:85–93. 26184049 10.1016/j.jconrel.2015.07.006

[12] Creighton S , Bourne TH , Lawton FG , Crayford TJB , Vyas S , Campbell S , et al. Use of transvaginal ultrasonography with color Doppler imaging to determine an appropriate treatment regimen for uterine fibroids with a GnRH agonist before surgery: a preliminary study. Ultrasound Obstet Gynecol. 1994;4:494–498. 12797131 10.1046/j.1469-0705.1994.04060494.x

[13] Reinsch RC , Murphy AA , Morales AJ , Yen SS . The effects of RU 486 and leuprolide acetate on uterine artery blood flow in the fibroid uterus: a prospective, randomized study. Am J Obstet Gynecol. 1994;170:1623–1627. discussion 1627‐8. 8203418

[14] Ma Y , Li G , Li J , Ren W‐d . The diagnostic value of superb microvascular imaging (SMI) in detecting blood flow signals of breast lesions: a preliminary study comparing SMI to color Doppler flow imaging. Medicine. 2015;94(36):e1502. 26356718 10.1097/MD.0000000000001502 PMC4616654

[15] Samanci C , Ozkose B , Ustabasioglu FE , Erol BC , Sirolu S , Yılmaz F , et al. The diagnostic value of superb microvascular imaging in prediction of uterine artery embolization treatment response in uterine Leiomyomas. J Ultrasound Med. 2021;40:2607–2615. 33599335 10.1002/jum.15647

[16] Wasserstein RL , Lazar NA . The ASA Statement on P‐Values: Context, Process, and Purpose. Am Stat. 2016;70:129–133.

[17] Lawless JF , Nadeau C . Some simple robust methods for the analysis of recurrent events. Technometrics. 1995;37:158–168.

[18] Zhan J , Diao XH , Jin JM , Chen L , Chen Y . Superb microvascular imaging‐a new vascular detecting ultrasonographic technique for avascular breast masses: a preliminary study. Eur J Radiol. 2016;85:915–921. 27130051 10.1016/j.ejrad.2015.12.011

[19] Lee YS , Kim MJ , Han SW , Lee HS , Im YJ , Shin HJ , et al. Superb microvascular imaging for the detection of parenchymal perfusion in normal and undescended testes in young children. Eur J Radiol. 2016;85:649–656. 26860680 10.1016/j.ejrad.2015.12.023

[20] Goeral K , Hojreh A , Kasprian G , Klebermass‐Schrehof K , Weber M , Mitter C , et al. Microvessel ultrasound of neonatal brain parenchyma: feasibility, reproducibility, and normal imaging features by superb microvascular imaging (SMI). Eur Radiol. 2019;29:2127–2136. 30315420 10.1007/s00330-018-5743-1 PMC6420458

[21] Lim AKP , Satchithananda K , Dick EA , Abraham S , Cosgrove DO . Microflow imaging: new Doppler technology to detect low‐grade inflammation in patients with arthritis. Eur Radiol. 2018;28:1046–1053. 29022101 10.1007/s00330-017-5016-4 PMC5811585

[22] Aleem FA , Predanic M . The hemodynamic effect of GnRH agonist therapy on uterine leiomyoma vascularity. Gynecol Endocrinol. 1995;9:253–258. 8540296 10.3109/09513599509160454

[23] Osuga Y , Enya K , Kudou K , Hoshiai H . Relugolix, a novel oral gonadotropin‐releasing hormone antagonist, in the treatment of pain symptoms associated with uterine fibroids: a randomized, placebo‐controlled, phase 3 study in Japanese women. Fertil Steril. 2019;112:922–929.e2. 31594635 10.1016/j.fertnstert.2019.07.013

[24] Seskute G , Montvydaite M , Butrimiene I . Power Doppler artifacts in evaluating inflammatory arthritis of small joints: comparison with a superb microvascular imaging technique. J Ultrasound. 2022;25:765–771. 10.1007/s40477-021-00643-2 35029838 PMC9402880

